# Cadaveric training model for the management of type B aortic dissection using thoracic endovascular aortic repair along with the PETTICOAT technique

**DOI:** 10.1016/j.jvscit.2025.101741

**Published:** 2025-01-18

**Authors:** Peter Osztrogonacz, Micah Thomas, Bahar Alasti, Rebecca Barnes, Alan B. Lumsden, Maham Rahimi

**Affiliations:** a Department of Cardiovascular Surgery, Houston Methodist Hospital, Houston, TX; b Department of Vascular and Endovascular Surgery, Semmelweis University, Budapest, Hungary; c Texas A&M College of Medicine, Bryan, TX; d McMaster University, Hamilton, ON, Canada

**Keywords:** PETTICOAT technique, Resident training, TEVAR, Type B aortic dissection, Type B aortic dissection cadaveric model, Vascular surgery techniques

## Abstract

This model gives residents and fellows practice in the surgical repair of a type B aortic dissection using thoracic endovascular aortic repair implantation by the Provisional Extension To Induce Complete Attachment (PETTICOAT) technique. A human cadaveric model was created to simulate the procedure. A Dacron graft was pulled into the aorta distal to the left subclavian artery using two glidewires. Imaging confirmed the creation of a second lumen by the graft positioned within the aorta to simulate an aortic dissection. The PETTICOAT technique was then implemented with the implantation of a Cook Dissection stent. This proved the method to be a reproducible means to train residents and fellows in type B aortic dissection repair using the PETTICOAT technique.

Numerous adjunctive management strategies exist for type B aortic dissection (TBAD) besides thoracic endovascular aortic repair (TEVAR), which is considered the primary surgical treatment option. However, the intricacy of these techniques can pose challenges for trainees in gaining proficiency due to limited training opportunities. This may be due to the emergent nature of the surgery, or the few circumstances that this case appears. The Provisional Extension To Induce Complete Attachment (PETTICOAT) technique has been pioneered by Nienaber et al,^1^ and it was suggested to be effective in the management of TBAD complicated with visceral malperfusion. The PETTICOAT technique utilizes a self-expandable bare metal stent deployed below the previously implanted stent graft to expand the true lumen at the level of the origin of the visceral arteries.

Based on other pulsatile flow models,^2^^,^^3^ the authors have developed a TBAD human cadaveric model to train vascular residents and trainees on the management of TBAD utilizing the PETTICOAT technique.

Our manuscript introduces a cadaveric model for type B aortic dissection, specifically designed to facilitate training in TEVAR implantation with the PETTICOAT technique, aimed at enhancing trainee proficiency in this approach.

## Methods

**Fig 1:**
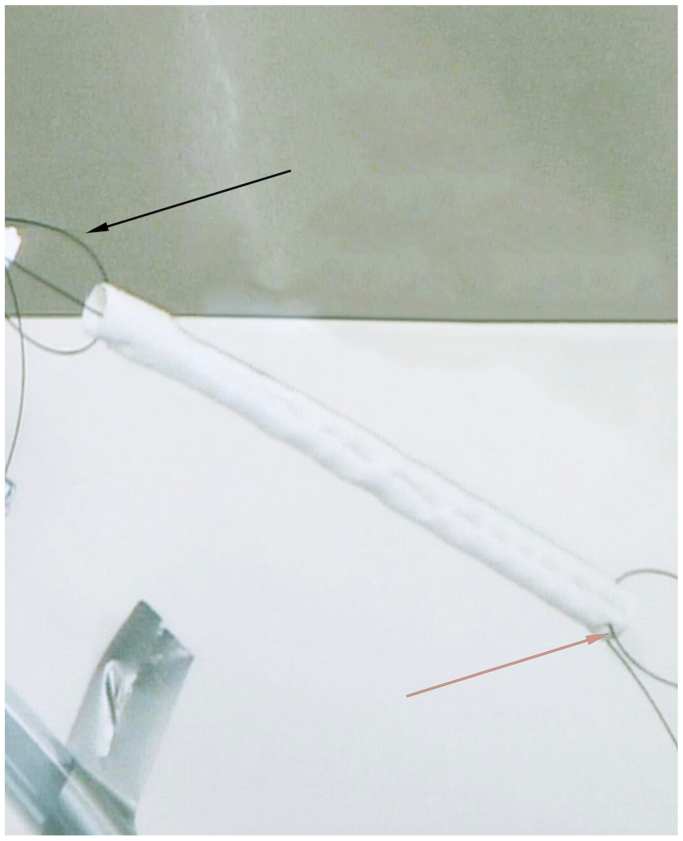
Image of the Dacron graft (DG) that is used to simulate a type B aortic dissection. Each *arrow* points to the glidewires inserted into the proximal and distal ends.

**Fig 2:**
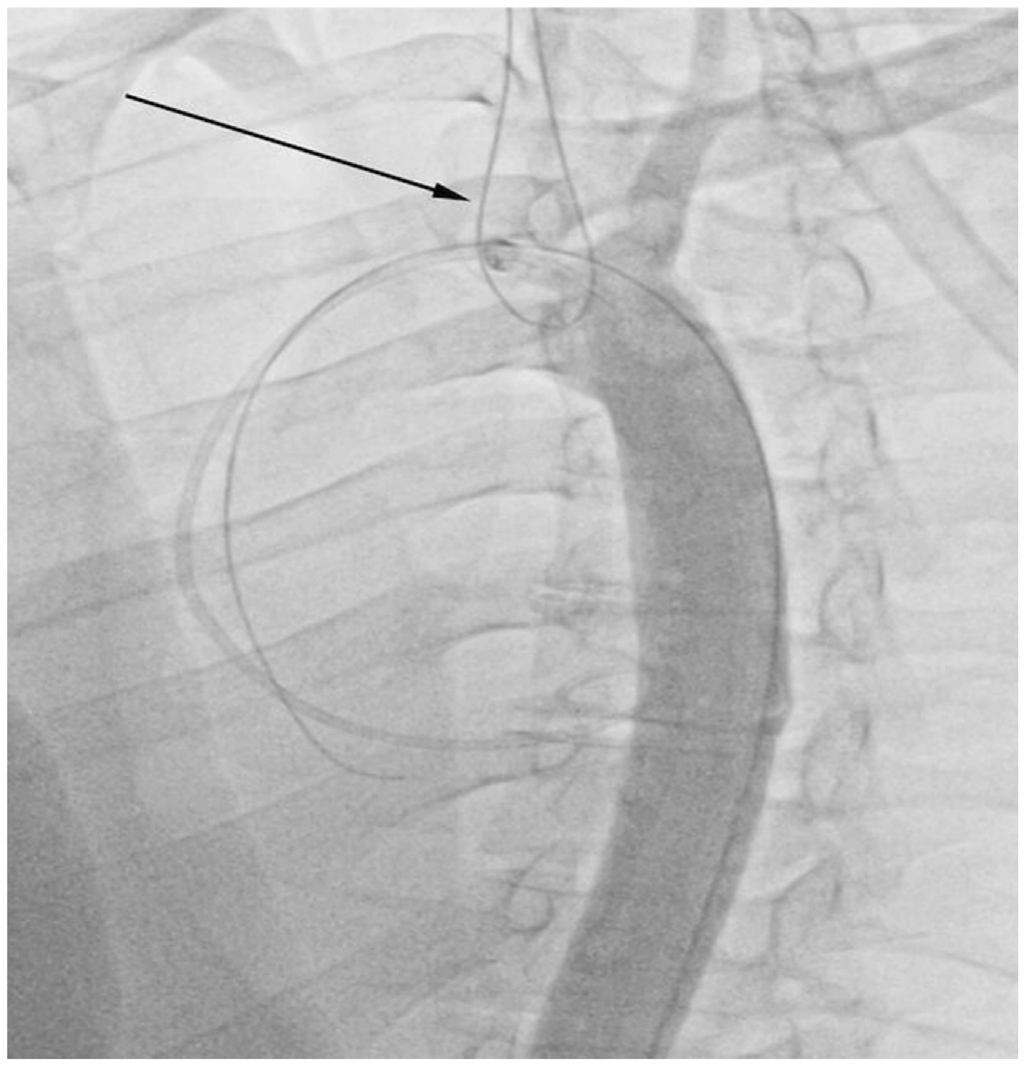
Angiographic image portraying the thoracic endovascular aortic repair (TEVAR) set up of the Dacron graft (DG). The *arrow* emphasizes the snare used to introduce the graft to the left common carotid artery.

**Fig 3:**
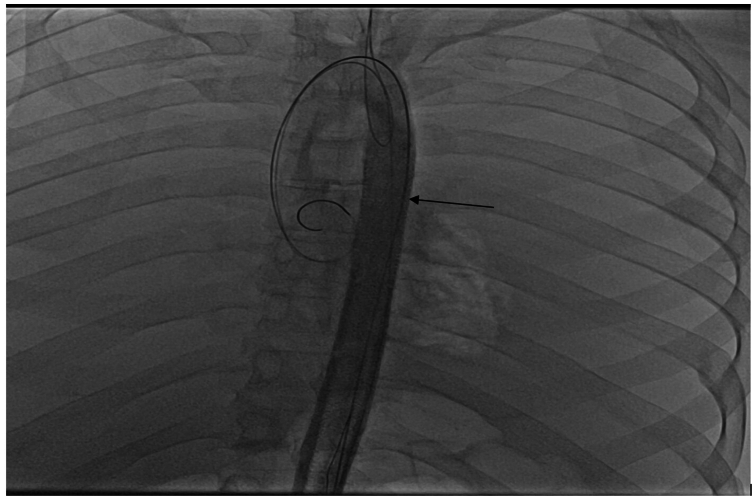
Angiographic image confirming the Dacron graft (DG) placement distal to the left subclavian artery. The *arrow* points to the DG placed within the aorta.

**Fig 4:**
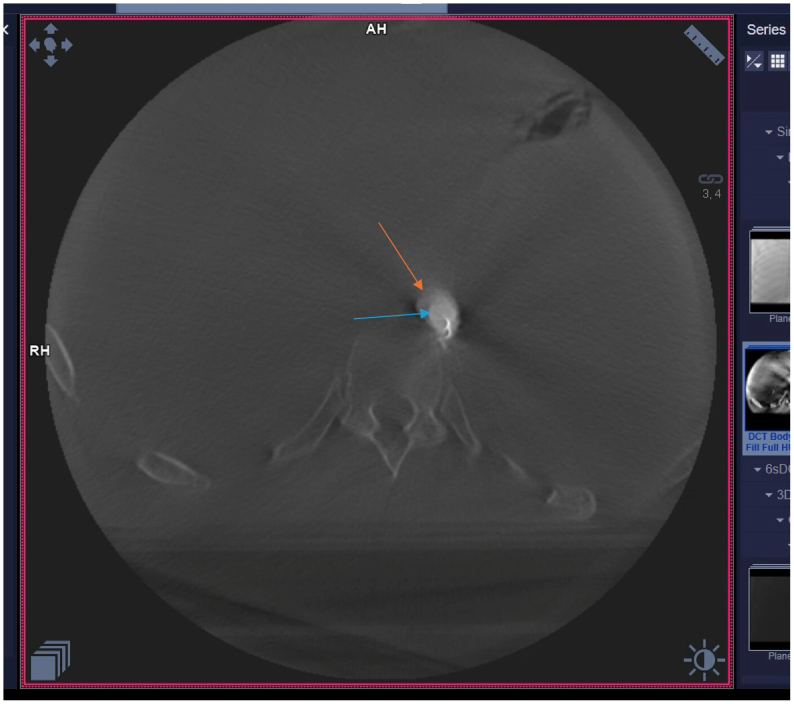
Intraoperative cone-beam computed tomography (CBCT) image portraying the creation of true and false lumens within the aorta. The *orange arrow* shows the false lumen of the aortic wall, and the *blue arrow* shows the true lumen made by the Dacron graft (DG) within the aorta.

**Fig 5:**
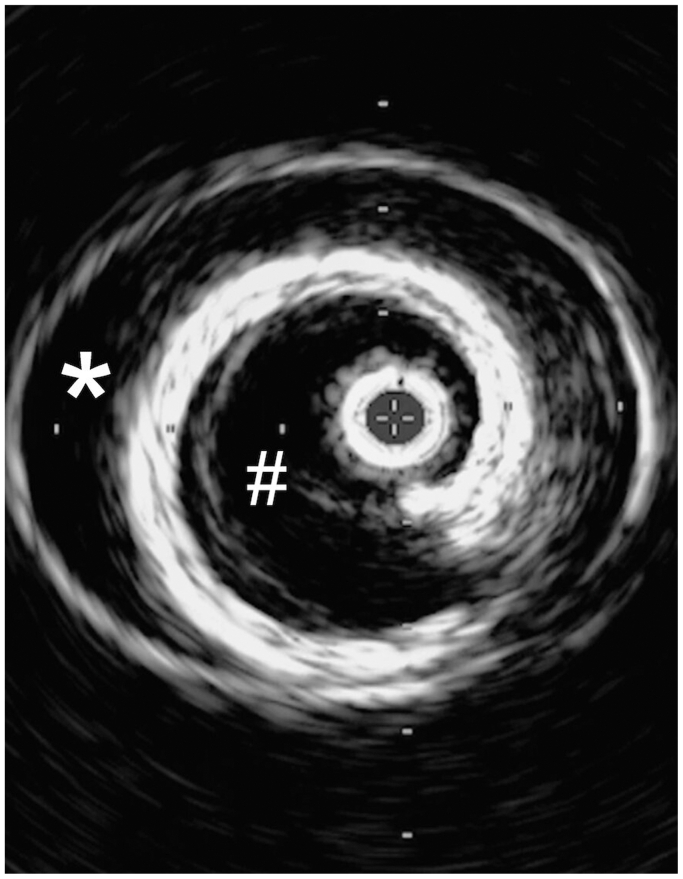
Intravascular ultrasound image portraying the creation of two lumens within the aorta. The *star* shows the false lumen of the aortic wall, whereas the *pound sign* marks the true lumen created by the Dacron graft (DG) within the aorta.

The study was conducted at the state-of-the-art training facility within Houston Methodist Hospital. A cadaveric pulsatile flow model was created with the cadaver placed supine on the operating table in the hybrid suite. The total cost associated with this project was $4000 ($1500 for the cadaver and $2500 for access to the hybrid suite). The materials used to simulate a type B aortic dissection and create the PETTICOAT technique were donated to the hospital. Access to the left common carotid artery and left common femoral artery was established, and sheaths were placed serving as inflow and outflow points for the perfusate. Flow was generated using an arthroscopic irrigation pump. A 40-cm long Dacron graft (DG) was prepared, leaving the proximal 10 cm intact, with longitudinal incisions made on the distal portion to serve as fenestration. This graft was measured to have a diameter approximately 20% smaller than the 15 mm diameter of the aorta. This technique utilizes the entire length of the DG. The simulated dissection may cover other vessels once in position to simulate the dynamic nature of a type B aortic dissection. The graft material was punctured proximally and distally to accommodate glidewires. This allows for the positioning of the graft (Fig 1). A glidewire was introduced into the aorta through the sheath in the left common carotid artery and externalized from the femoral sheath using a snare (Fig 2). The wire was then guided through the proximal puncture sites of the DG and fed back to the femoral sheath, externalizing once more from the carotid sheath using a snare. A second glidewire was introduced through the distal puncture holes on the DG. The graft was then pushed through the femoral sheath and simultaneously pulled from the wires externalized at the carotid artery. The DG was positioned just distal to the left subclavian artery to create the true and false lumens to complete the setup (Fig 3). Intraoperative cone-beam computed tomography (CBCT) (Fig 4) and intravascular ultrasound (Fig 5) confirmed two distinct lumens within the aorta—a true lumen within the DG and a false lumen outside.

## Results

A cadaveric model was created with a DG to simulate a true and a false lumen to enhance training in management of acute TBAD cases. An Amplatz wire facilitated the introduction of a Gore CTAG stent graft just distal to the subclavian artery for treatment of the created aortic dissection. The PETTICOAT technique was implemented by implanting a Cook Dissection stent distally to the stent graft towards the visceral portion of the aorta. Completion angiography confirmed perfusion of the visceral arteries.

## Discussion

Many methods to recreate TBAD in a cadaver were attempted before creating our above-discussed model with the PETTICOAT technique. This is because aortic dissection creation in a cadaveric setting is time-consuming and has a high failure rate, which leads to an inconsistent dissection morphology. Additionally, aortic dissection creation in animal models has a high failure rate,^4^, ^5^, ^6^ and reproducibility remains an issue with this type of approach.

Our objective was to construct a model that circumvents substantial technical hurdles by prioritizing time, efficiency, and reproducibility. This model proved to meet these criteria as it has since been recreated three times with the deployment of an undersized DG within the aorta. The graft utilized in this model was 20% smaller than the aorta of the cadaver. This facilitates the differentiation between true and false lumens and subsequent procedural interventions within these distinct luminal spaces. Alternatives such as utilizing cadaveric or swine inferior vena cava were considered; however, Dacron was selected due to its widespread availability, ease of delivery, and reduced setup duration. Although efforts were made to secure the proximal segment of the DG to the descending thoracic aorta using endoanchors, cost considerations and inadequate fixation deterred its implementation. Nevertheless, the proximal and distal loops effectively anchored the DG in place for device insertion into the true and false lumens, respectively. The proximal Dacron segment simulated the diseased region of the aorta, with plans to position the device 20 mm proximal to this zone. The lumen within the DG represented the true lumen, while the space between the graft and the native aortic wall symbolized the false lumen.

Cadaveric models afford opportunities for: (1) conducting CBCT scans to inform procedural planning; (2) obtaining arterial access (either percutaneously or through open exposure); (3) acquiring direct feedback and handling experience with various devices across diverse anatomic scenarios; (4) selecting and delivering devices; and (5) performing intravascular ultrasound. Although the lifelike nature of cadaveric models may introduce challenges in straightforward tasks, as compared with virtual simulators, the incorporation of the DG contributes to a potentially replicable and readily implementable framework.

Although our model boasts ease of setup and reproducibility, it is not devoid of limitations. These constraints encompass the expenses associated with model creation, the availability of devices and cadavers, and the uncertainty regarding the aortic diameter of cadavers prior to model construction. Addressing small aortic diameters identified on CBCT scans may involve deploying undersized Dacron grafts and utilizing smaller devices. As a default recommendation, a 12 mm DG is advised for an average-sized aorta. Challenges arising from model costs and access to resources could be mitigated through the organization of annual training programs hosted by regional centers equipped with the requisite infrastructure to support such endeavors. Concerning cadaver selection, preference is given to males with normal body mass index due to their typically larger aortic diameter compared with females. Normal body mass index individuals are favored over overweight counterparts to optimize image quality at equivalent levels of radiation exposure.

Complicated TBAD represents one of the most challenging aortic pathologies a vascular surgeon could face during their career. Thorough knowledge and an up-to-date skillset are required to tackle this treacherous aortic condition. The PETTICOAT technique provides a useful adjunct to TEVAR when visceral malperfusion complicates a TBAD case.

Our proof-of-concept model allows for practice both in TEVAR and the PETTICOAT technique in a safe and lifelike environment. The use of a DG to simulate the true lumen contributes to a high degree of reproducibility, which could further enable consistent scaling across the board.

## Conclusion

Our research establishes the feasibility of creating a TBAD model suitable for performing TEVAR with the PETTICOAT Technique. The use of a DG for false and true lumen separation potentially allows for a high reproducibility rate.

## Funding

This work was completely funded by 10.13039/100015190Houston Methodist Hospital. Houston Methodist had no involvement in the study design or collection, analysis, and interpretation of data. Houston Methodist was not involved in the decision to submit the manuscript for publication.

## Disclosures

None.
